# Supplementary material for: Influenza virus genome reaches the plasma membrane via a modified endoplasmic reticulum and Rab11-dependent vesicles
Source: Nat Commun. 2017 Nov 9;8:1396. doi: 10.1038/s41467-017-01557-6 (PMC5680169; doi:10.1038/s41467-017-01557-6)
Supplement: Supplementary file 1 — Supplementary Information [file 41467_2017_1557_MOESM1_ESM.pdf]

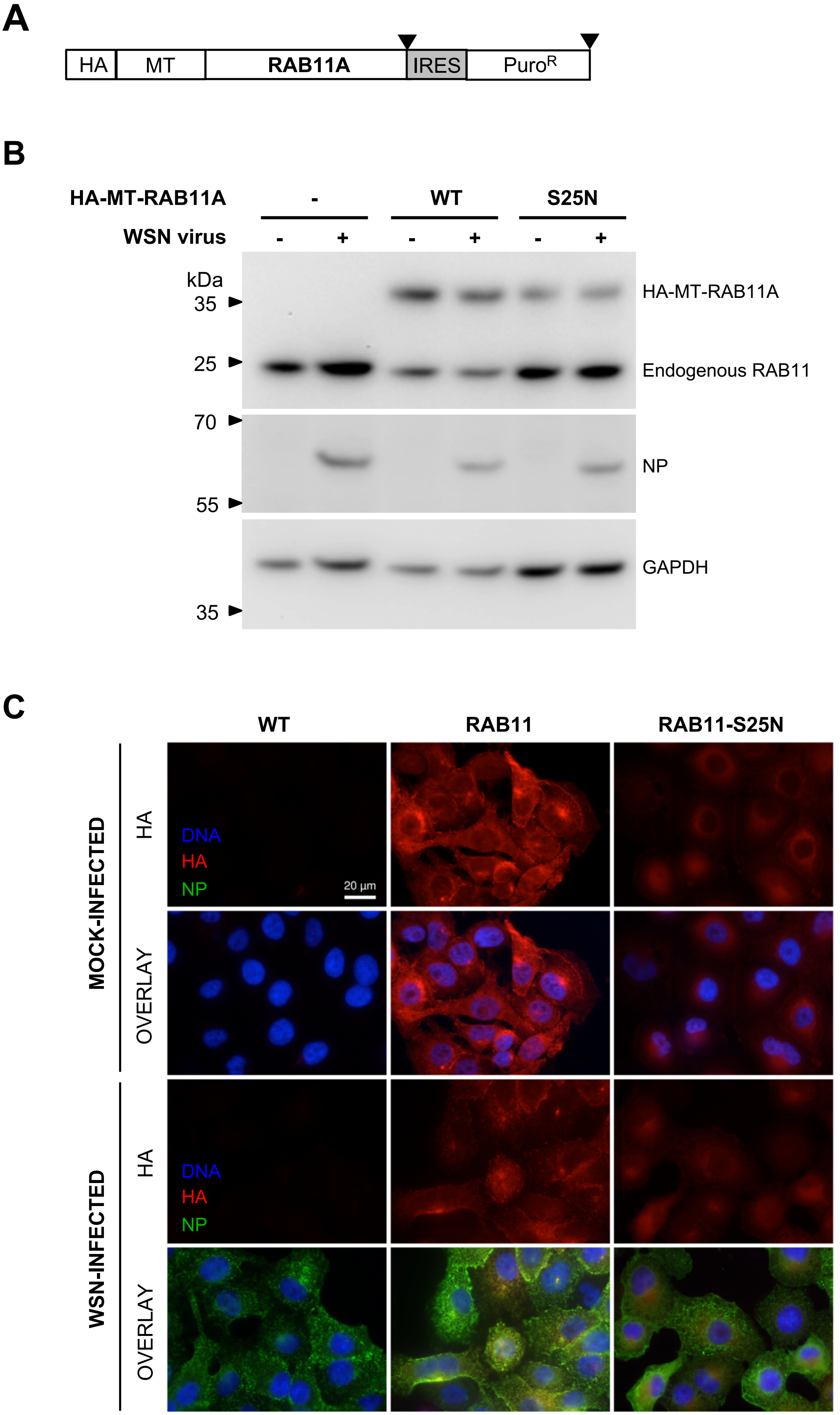
A
HA
MT
RAB11A
IRES
PuroR
B
HA-MT-RAB11A
-
WT
S25N
WSN virus
- + - + - +
kDa
35
25
70
55
35
HA-MT-RAB11A
Endogenous RAB11
NP
GAPDH
C
WT
RAB11
RAB11-S25N
MOCK-INFECTED
WSN-INFECTED
HA
OVERLAY
DNA
HA
NP
20 µm

Supplementary Figure 1. Cell lines expressing HA-MT-Rab11 or HA-MT-Rab11-S25N.
(A) Schematic representation of the Rab11A expression cassette. The HA tag and metallothionein tag (MT) were inserted upstream the Rab11A open reading frame into the bicistronic expression vector pIRES-puro3. The presence of an EMCV internal ribosome entry site (IRES) between the Rab11 and the puromycin-N-acetyl-transferase ($Puro^R$) sequences allows to exert a selective pressure on the entire expression cassette by adding puromycin in the culture medium upon transfection of the plasmid. Stop codons are indicated by an arrowhead.
(B) Western-blot detection of the endogenous and recombinant Rab11 proteins in the stable HA-MT-Rab11- or HA-MT-Rab11-S25N-expressing A549 cell lines. Cells were either mock-infected (-) or infected with the WSN virus (+) and cell lysates were prepared at 8 hpi. The membrane was re-hybridized with an anti-NP and an anti-GAPDH antibody for viral infection control and loading control, respectively.
(C) Indirect immunofluorescence detection of the recombinant Rab11A protein in the stable HA-MT-Rab11- or HA-MT-Rab11-S25N-expressing A549 cell lines, either mock-infected or infected with the WSN virus. Cells were fixed at 8 hpi and stained for HA-Rab11 (red) and NP (green).

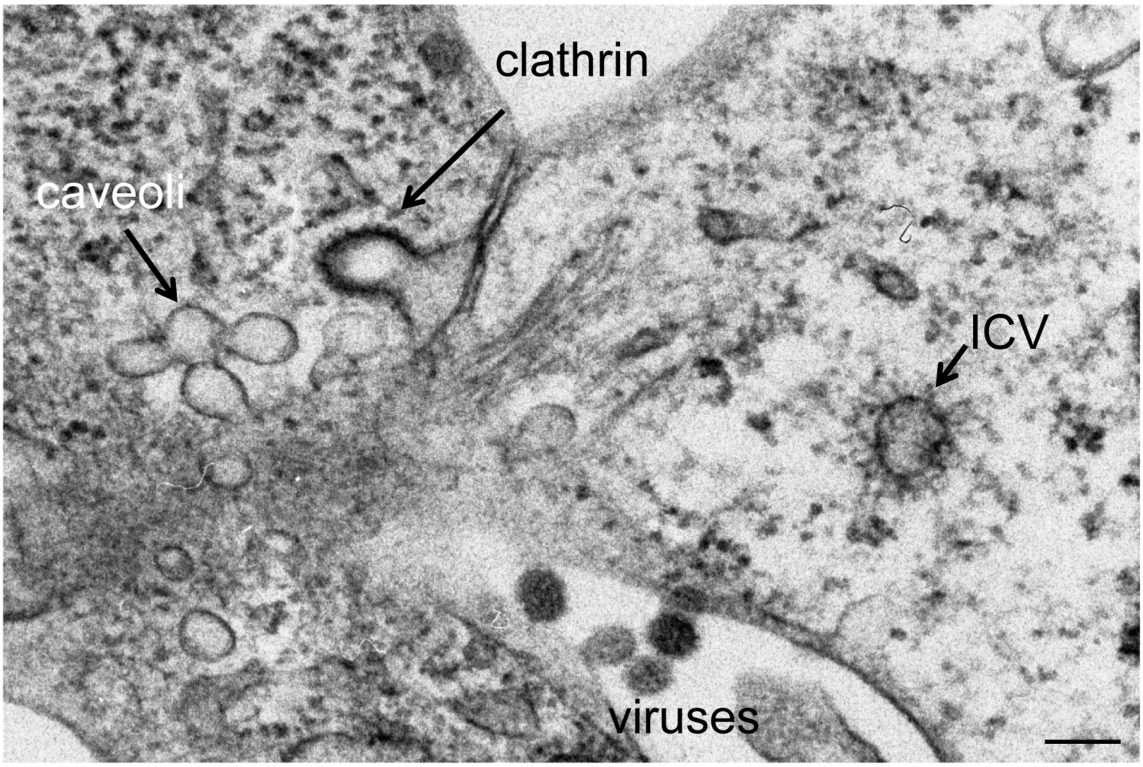

Supplementary Figure 2. ICVs are morphologically distinct from other transport vesicles.
Ultrathin section of an IAV-infected A549 cell at 8 hpi showing a coated pit with the characteristic clathrin coat, caveoli, and an ICV. Extracellular viruses are also marked.
Scale bars, 100 nm.

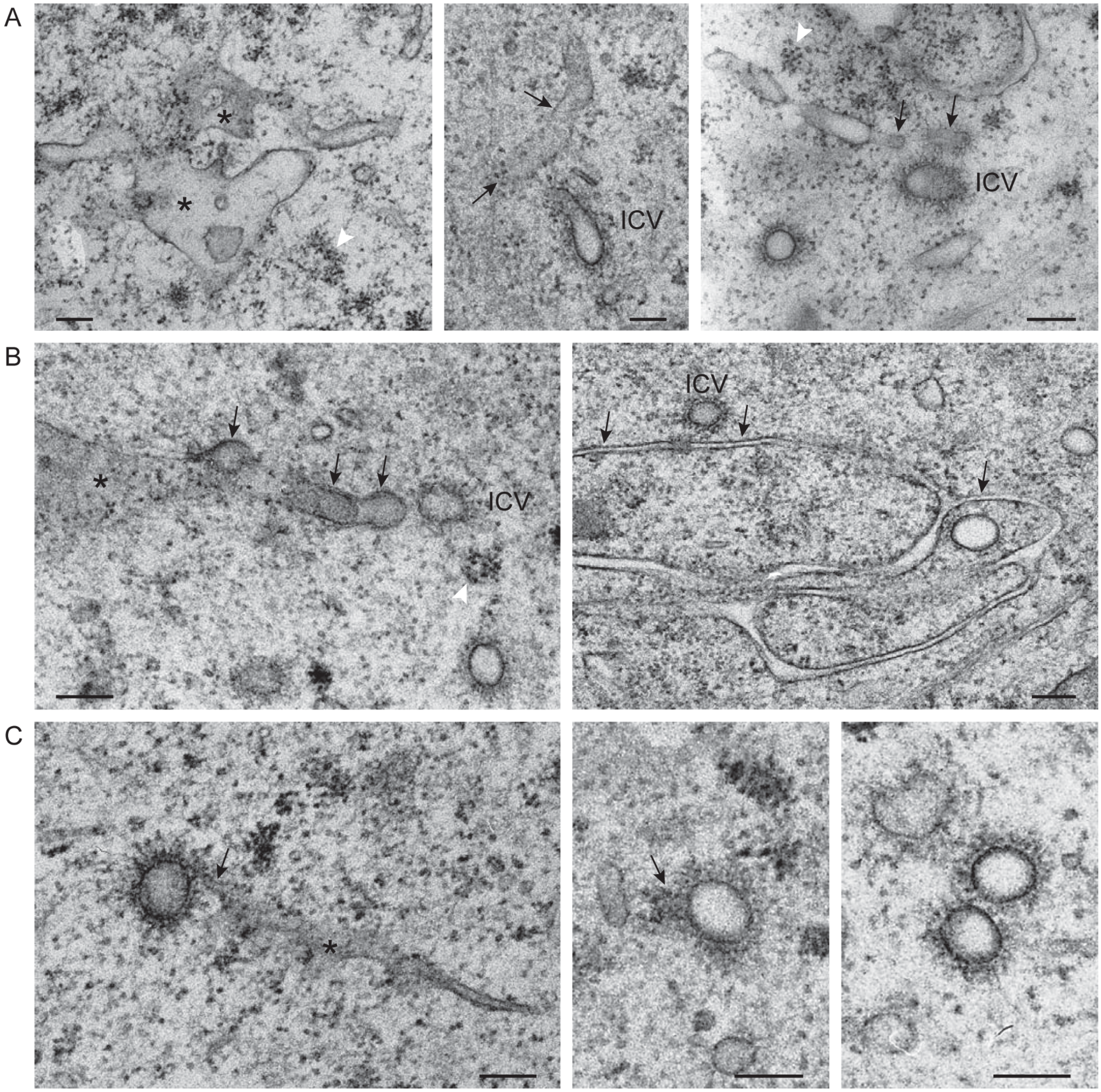

Supplementary Figure 3. Collection of (extra) images showing ER remodelling (A and B) and ICV assembly and structure (C) at 8 hpi.
(A) and (B) Swollen ER cisternae (asterisks) are surrounded by groups of ribosomes (white arrowheads). Black arrows point to remodelled ER cisternae in contact or close to ICVs.
(C) The irregular coat of ICVs is shown in detail at high magnification.
Scale bars, 200 nm.

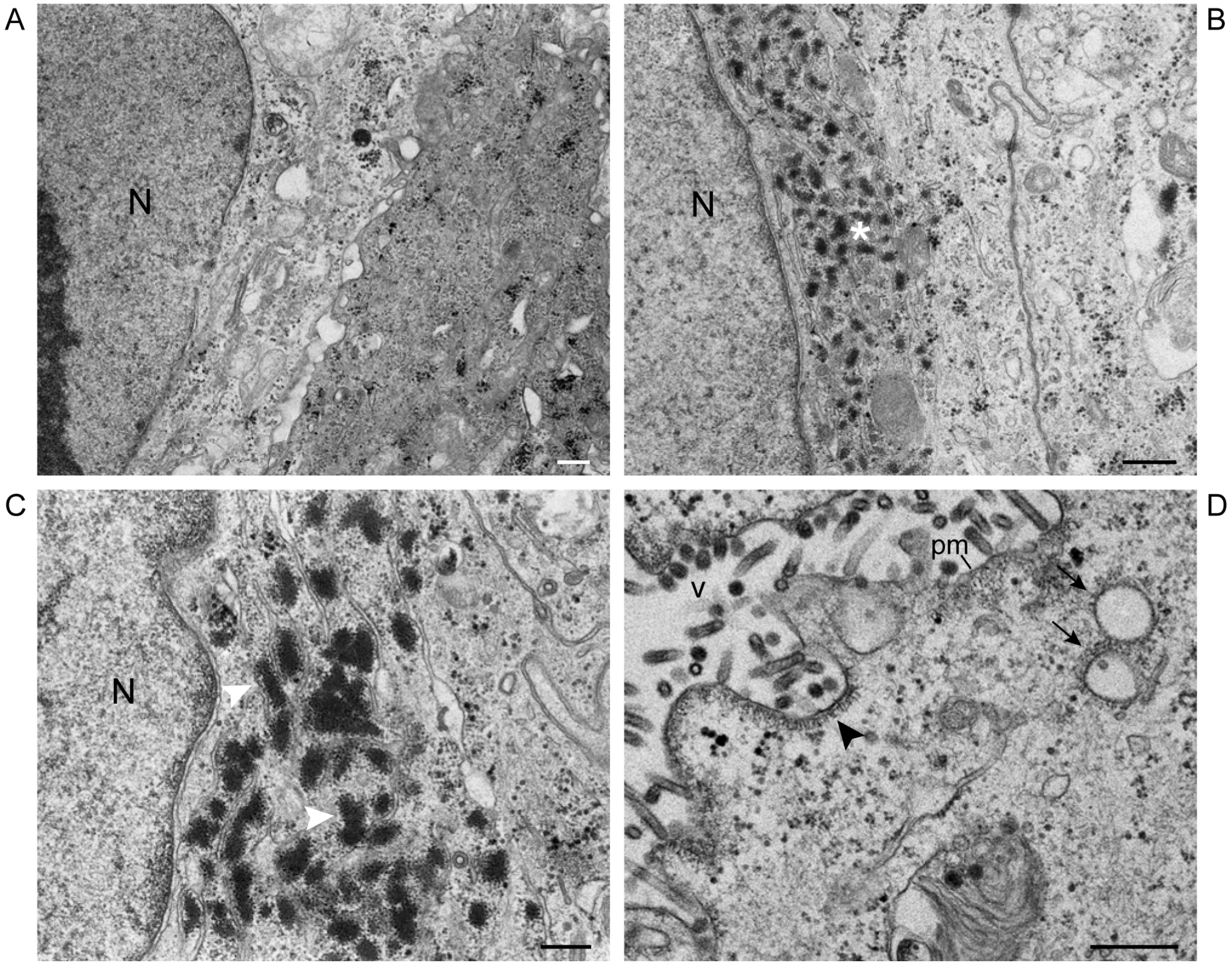

Supplementary Figure 4. Influenza virus infection induces remodeling of ER and ICVs in Calu3 cells.

(A and B) Perinuclear regions of mock- and WSN-infected Calu-3 cells, respectively as seen by TEM of ultrathin-sections. Images show (A) a random distribution of organelles in mock-infected cells and (B) recruitment of ER and groups of ribosomes in WSN-infected cells at 14 hpi (asterisks). N, nucleus.

(C) Close-up of a perinuclear region of a WSN-infected Calu-3 cell showing altered ER membranes and groups of ribosomes (arrowheads).

(D) ICVs (arrows) near a virus budding region at the plasma membrane (pm). The irregular coat in ICVs is very similar to the small structures under the plasma membrane (arrowhead). V, viruses. Scale bars, 0.5 μm.

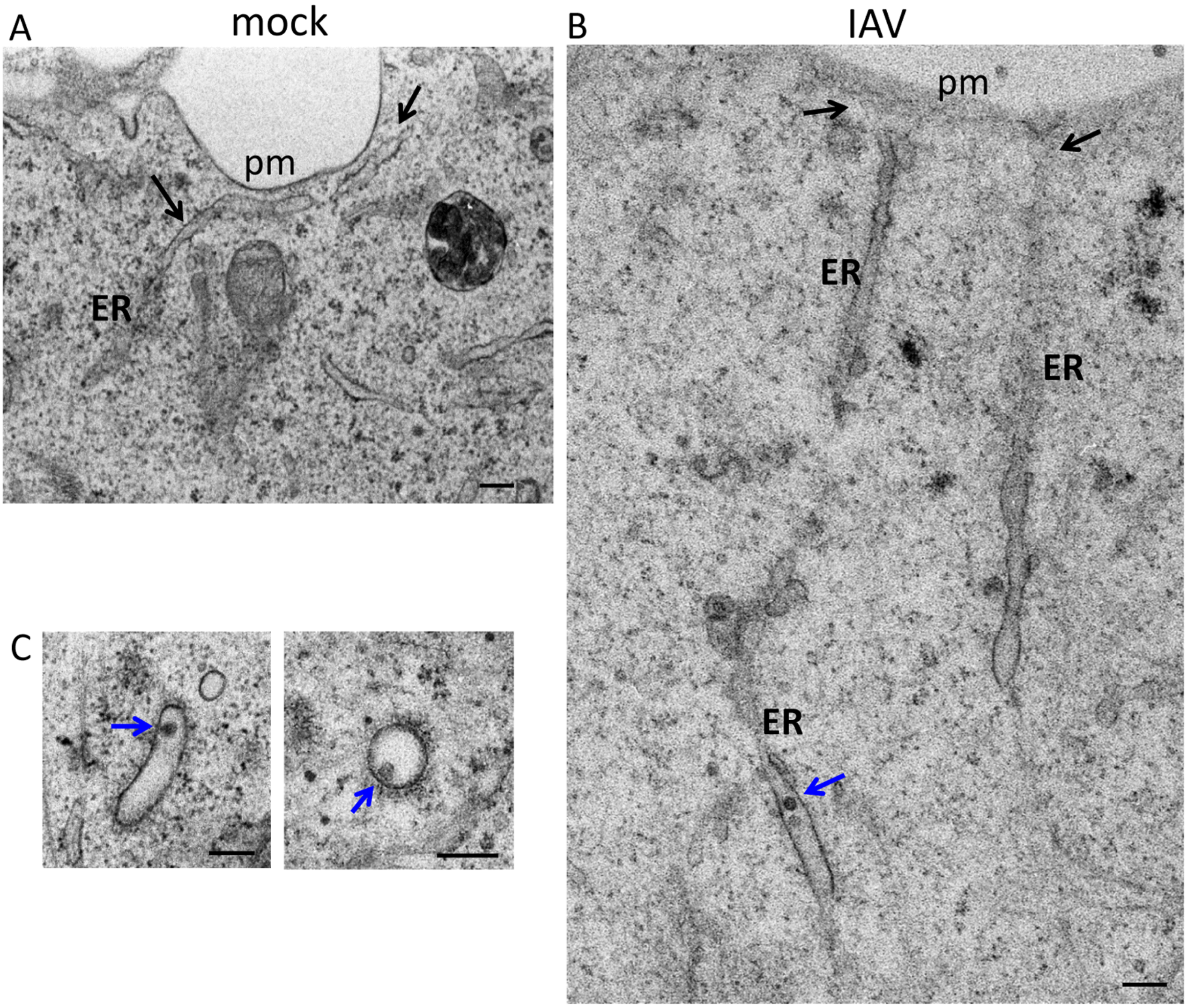

Supplementary Figure 5. ER cisternae close to the plasma membrane in mock- and IAV-infected cells at 8 hpi.
(A) In mock-infected cells, ER cisternae close to the cell surface (black arrows) are oriented in parallel to the plasma membrane (pm). This was observed in 13 out of 25 cells.
(B) An IAV-infected cell with ER cisternae close to the cell surface (blue arrows) and perpendicular to the plasma membrane. This observation was made in 25 out of 25 cells. Vesicles are often seen inside ER cisternae (blue arrow).
(C) Tubular and spherical ICVs with internal vesicles (blue arrows) similar to those in the ER lumen.
Scale bars, 200 nm.

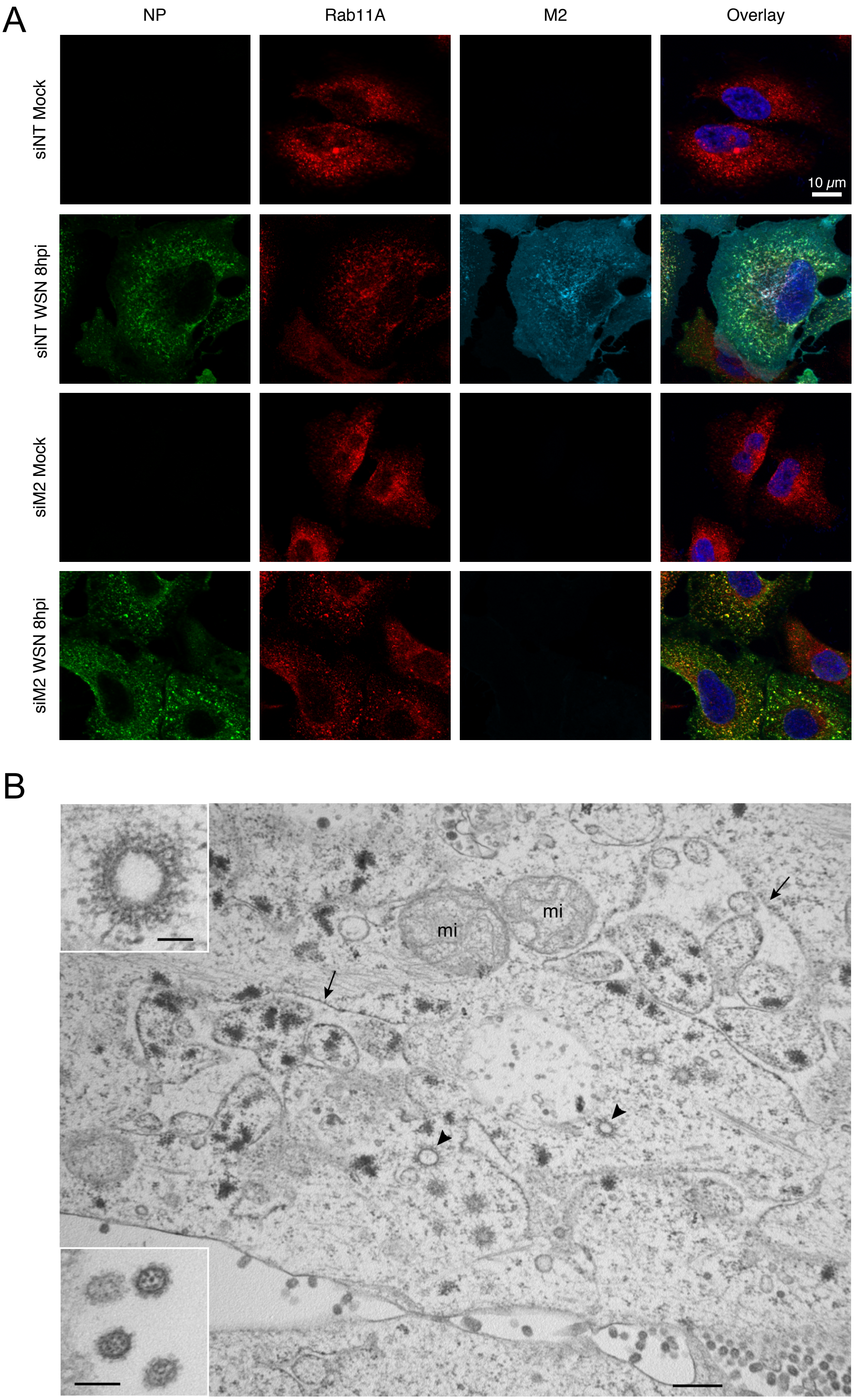
A
NP
Rab11A
M2
Overlay
siNT Mock
siNT WSN 8hpi
siM2 Mock
siM2 WSN 8hpi
10 μm
B
mi
mi

Supplementary Figure 6. M2 is not requested for ER remodeling and ICV formation. A549 cells were transfected with siRNA specific for IAV M2 protein, infected with WSN and processed at 8 hpi for (A) indirect immunofluorescence staining for M2, NP and Rab11, and (B) for TEM. In (B), mainfield shows characteristic ER remodeling (arrows) and ICVs (arrowheads). Inset on the top is an ICV at high magnification. Inset on the bottom shows viral particles.
Scale bars, 500 nm for mainfield; 100 nm for insets.

A

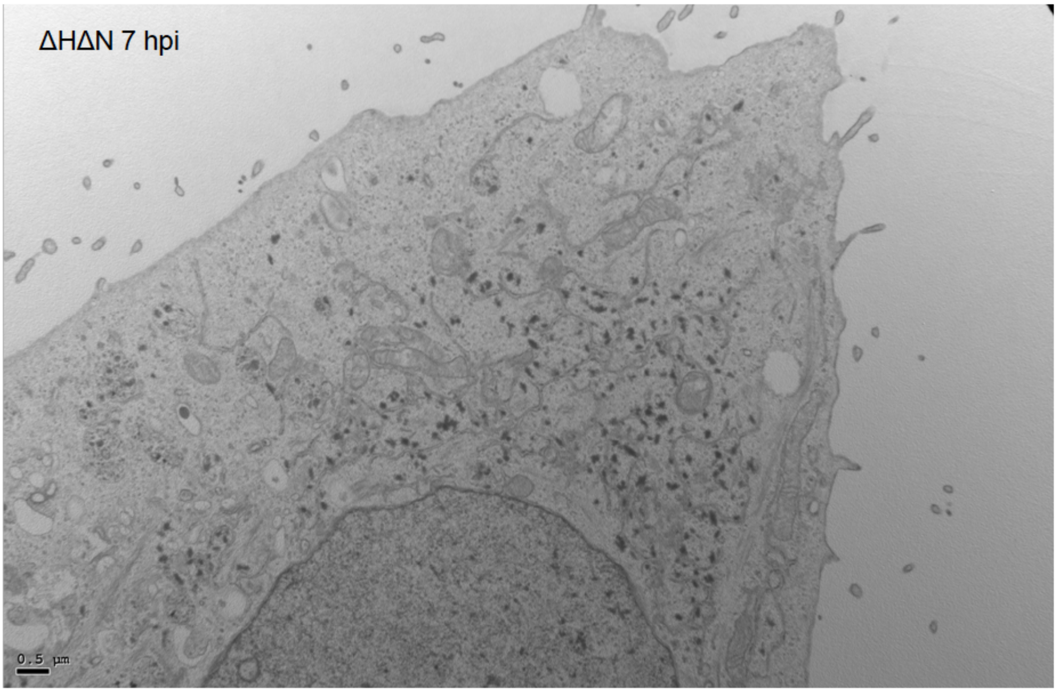

B

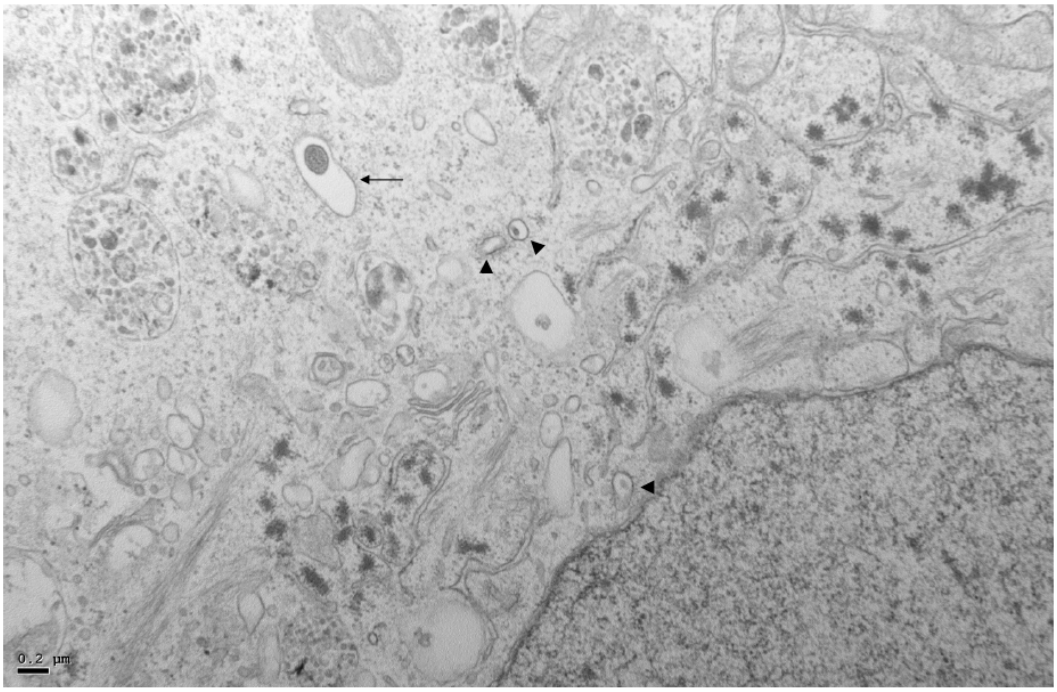

Supplementary Figure 7. Infection with a WSN virus which does not express HA nor NA still induces ER remodeling and ICV formation. (A) and (B) A549 cells were infected with an HA(VSVG)NA(mCherry) recombinant WSN virus and processed at 7 hpi for TEM of ultrathin sections. Images at two different magnifications are shown. In (B) ICVs of standard size (arrowheads) and of larger size (arrow) are shown.

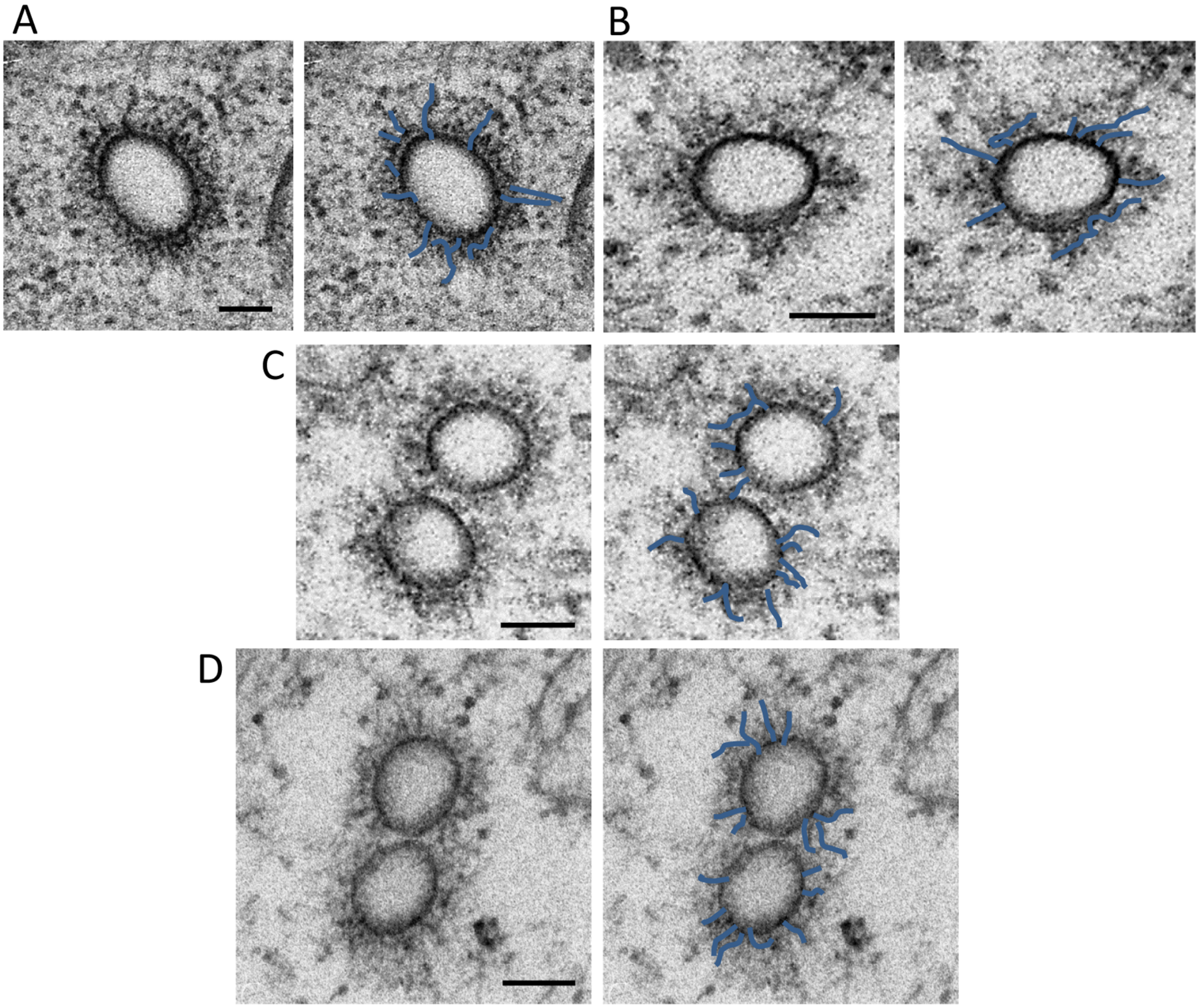

Supplementary Figure 8. ICVs' filaments have the same length as IAV vRNPs. Measurement was done on ultrathin sections of IAV-infected cells. 137 filaments from 16 ICVs were measured.
(A to D) Images show 6 ICVs included in this study. Selected filaments are marked with blue lines in the left panels. Scale bars, 100 nm.

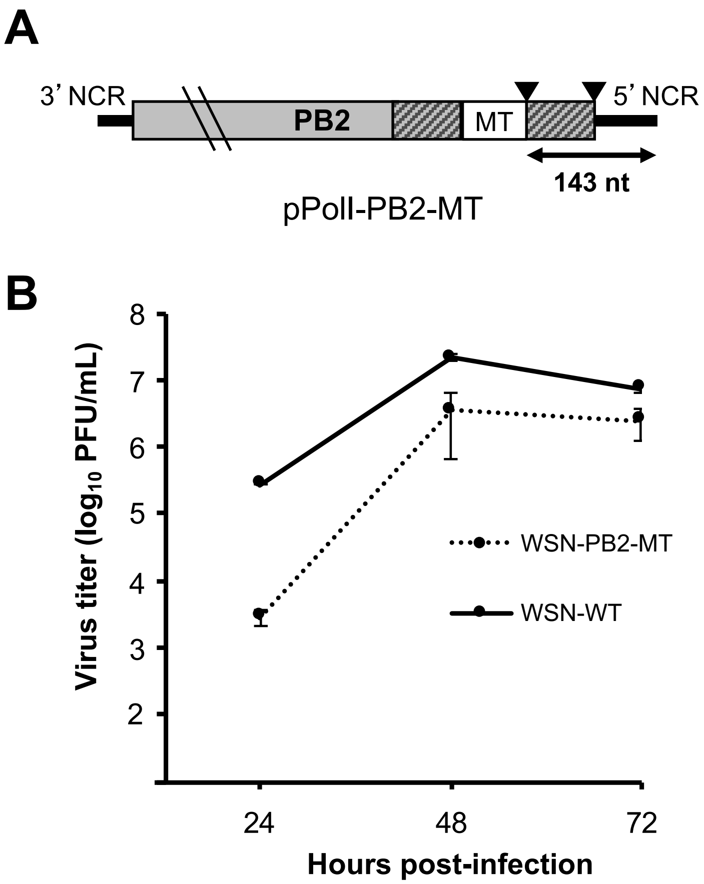

Supplementary Figure 9. The recombinant WSN virus encoding a PB2-MT protein.

(A) The vRNA encoding a PB2-MT fusion protein is represented schematically. The PB2 open reading frames (light grey box) is fused to the sequences encoding the MT protein (white box) and flanked by the 3' and 5' viral non-coding regions (NCR, thick lines). The MT and PB2 sequences are separated with an Ala-Ala-Ala-Gly-Gly linker. The region of the PB2 coding sequence that is duplicated is represented as hatched boxes. Stop codons are indicated by an arrowhead. The length of the sequence kept identical to the wild-type at the 5' extremity of the genomic segment is indicated by a double-headed arrow.

(B) A549 cells were infected with the wild-type (solid line) or the PB2-MT (dotted line) WSN virus at a moi of 0.001 pfu/cell. At the indicated times post-infection the viral titers were determined by plaque assay on MDCK cells. The results are expressed as the mean ± SD of triplicates.

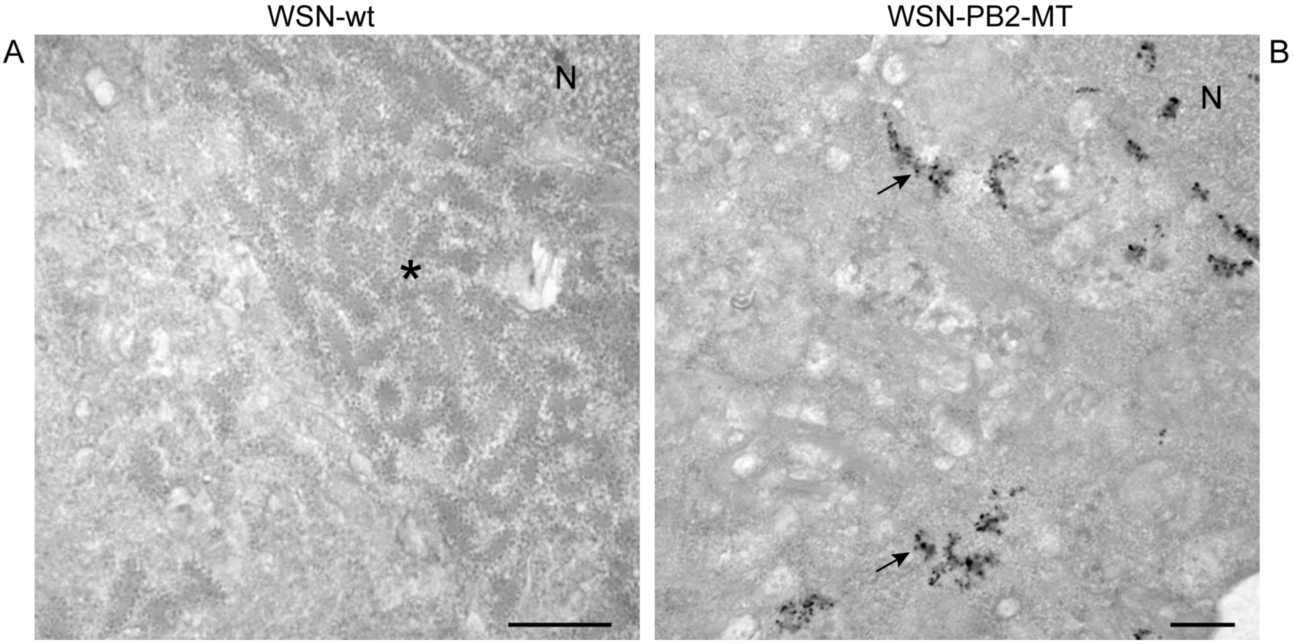

Supplementary Figure 10. METTEM detection of vRNPs-MT-gold-silver is a highly specific method.

(A) A549 cell infected with the wild-type WSN virus and incubated with gold and silver at 8 hpi before embedding, thin-sectioning and TEM. The nucleus (N) and virus-induced modified ER (asterisk) are free of label.

(B) A549 cell infected with the WSN-PB2-MT virus and incubated at 8 hpi with gold and silver before processing. Signals reveal the location of vRNPs in different compartments. Arrows point to labeled vRNPs. Scale bars, 0.5 µm.N, nucleus.

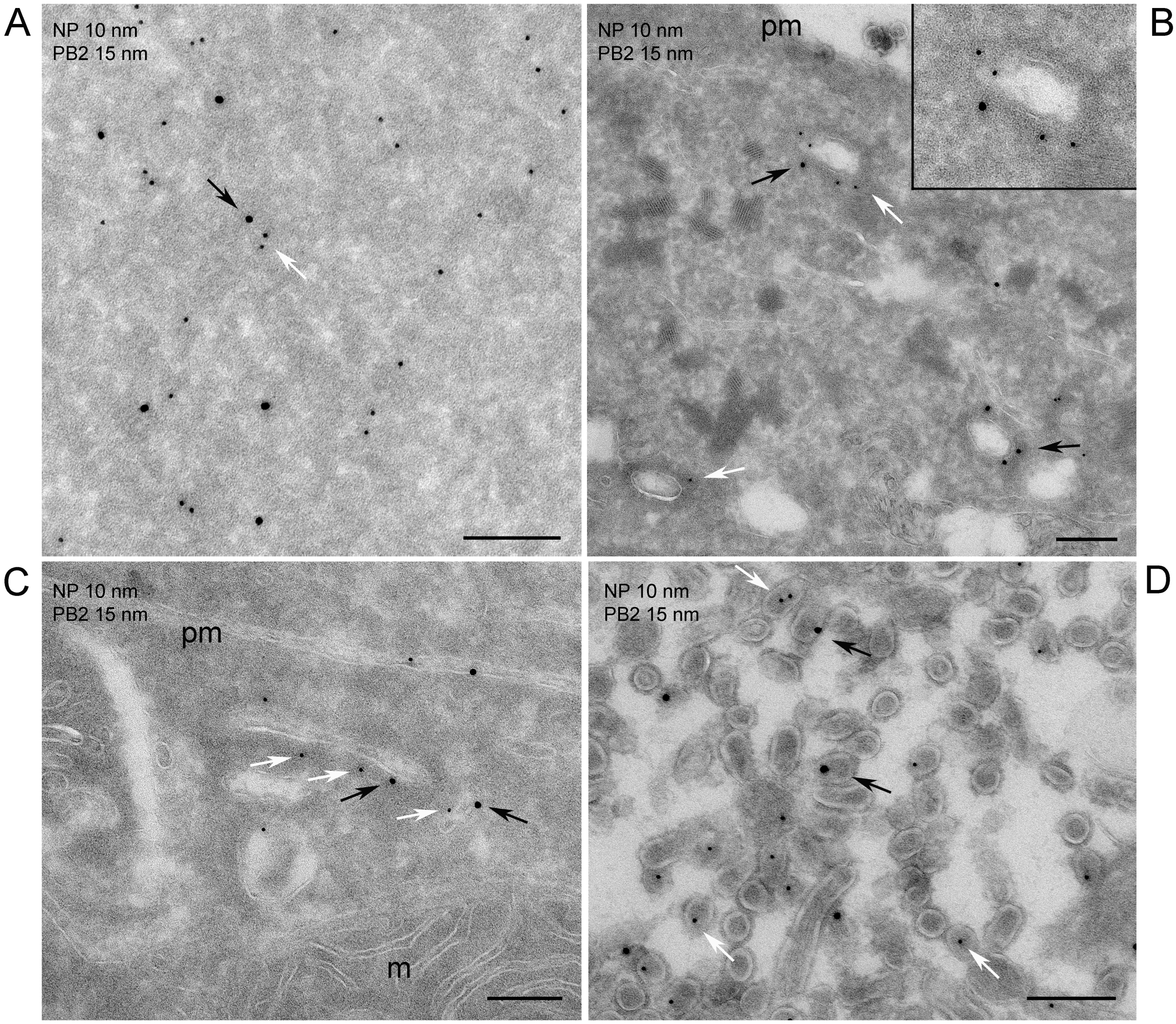

Supplementary Figure 11. NP and PB2 are present at the rough endoplasmic reticulum**.**
A549 cells were infected with the WSN virus and fixed for immuno-EM at 16 hpi. Thawed cryosections were labelled for NP (10 nm gold) and PB2 (15 nm gold). (A) Both proteins are present inside the nucleus, occasionally in close proximity (black and white arrow). (B) In the cytoplasma ICVs show can show label for NP or PB2 only (white and black arrow, respectively) but vesicles show also label for both proteins (black and white arrows). The insert shows a higher magnification of such an ICV. (C) NP and PB2 localize as well on tubular profiles with an irregular coat (black and white arrows). (D) Cross sections of viral particles. Either NP (white arrows) or PB2 (black arrows) are detected inside the viral particle. pm, plasma mebrane; m, mitochondria; scale bars, 200 nm.

A

B

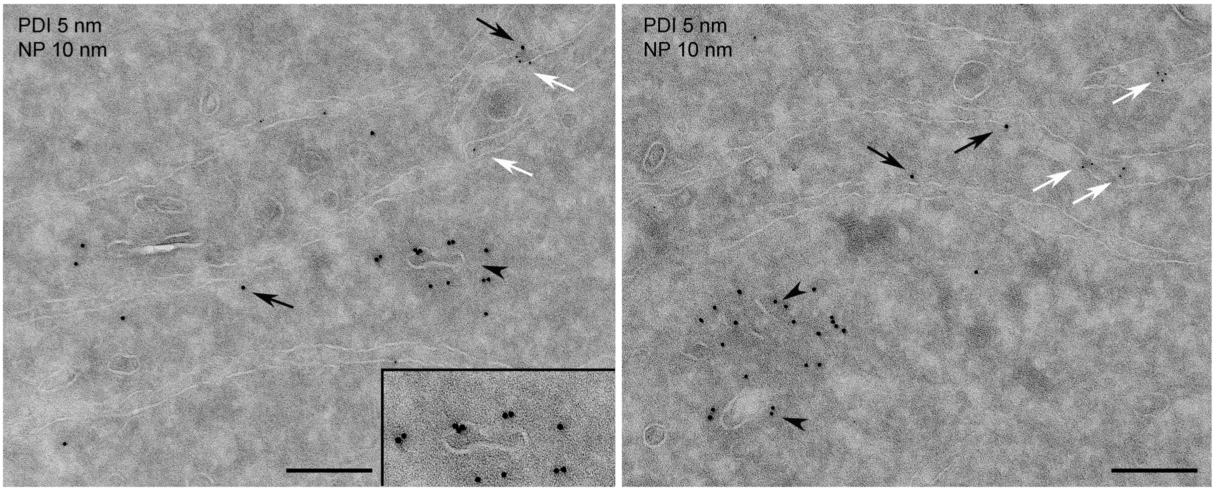

Supplementary Figure 12. NP is present at the rough endoplasmic reticulum. A549 cells were infected with the WSN virus and fixed for immuno-EM at 16 hpi. Thawed cryosections were labelled for PDI (5nm gold) and NP (10nm gold). (A-B) NP labeling can be found on ER cisternae (black arrows), which are characterized by the presence of PDI (white arrows). By contrast, ICVs (black arrowhead) show signal for NP as well but are devoid of label for PDI. The insert shows a zoom of an ICV. Note the amount of label for NP is higher on the ICVs compared to the ER cisternae. Scale bars: 200 nm.

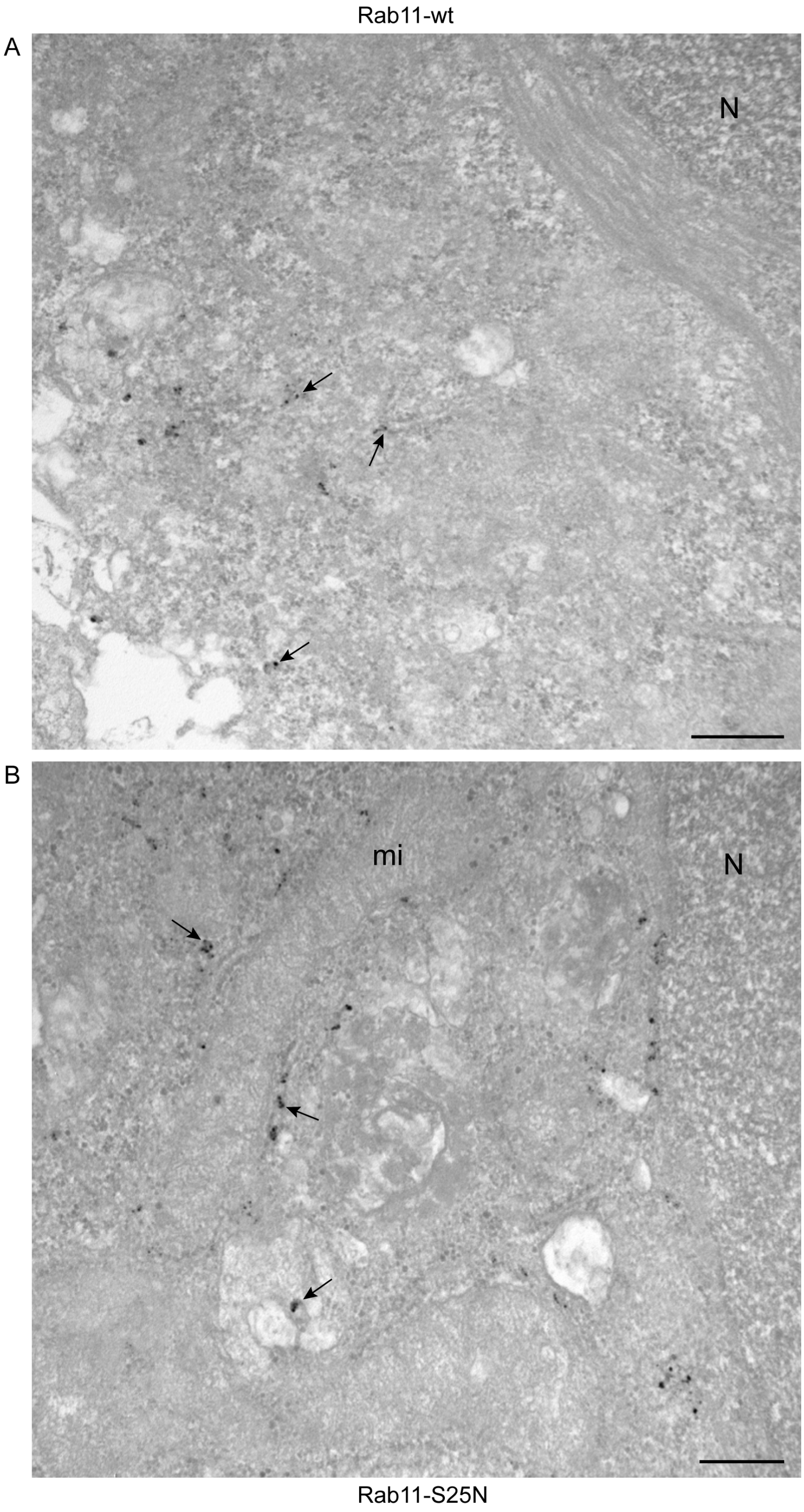

Supplementary Figure 13. Detection of HA-MT-Rab11 (A) and HA-MT-Rab11-S25N (B) in mock-infected cells by METTEM. Signals reveal the presence of these proteins in small vesicles and ER membranes (arrows); N, nucleus; mi, mitochondrion. Scale bars, 0.5 µm.

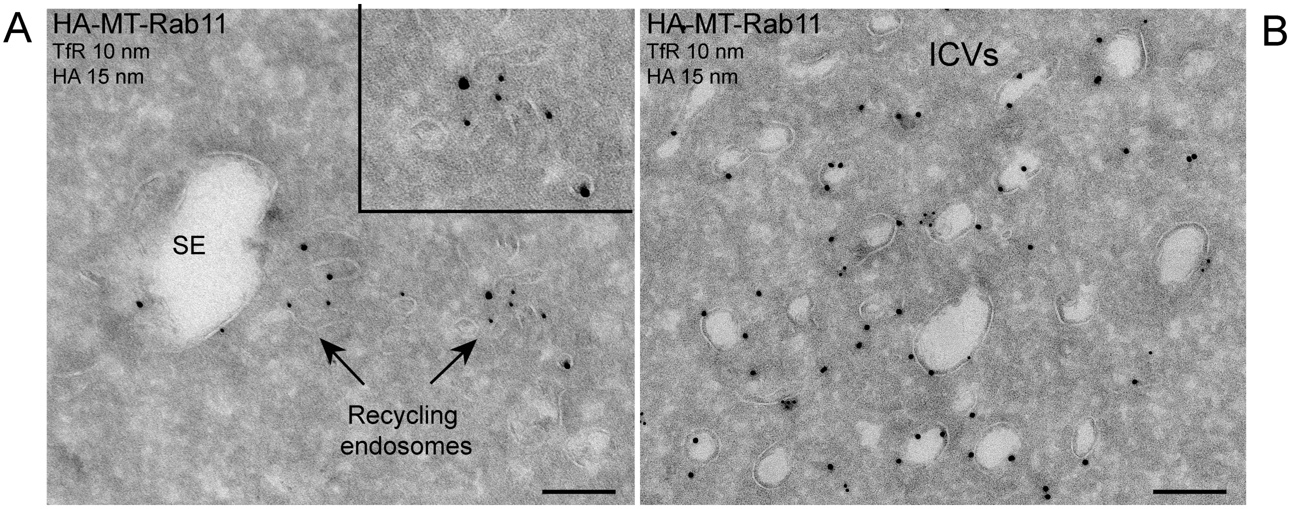

Supplementary Figure 14. ICVs are a new type of Rab11-positive vesicles distinct from recycling endosomes.

A549 cells expressing HA-MT-Rab11 were infected with the WSN virus and fixed for immuno-EM at 16 hpi. Thawed cryosections were labelled for HA (15 nm gold particles) and for Transferrin Receptor (TfR, 10 nm gold particles) that is a marker of recycling endosomes.

(A) The characteristic morphology of recycling endosomes is shown. SE, sorting endosome.

(B) ICVs show strong label for HA but are mostly devoid of TfR-associated label. Scale bars, 200 nm.

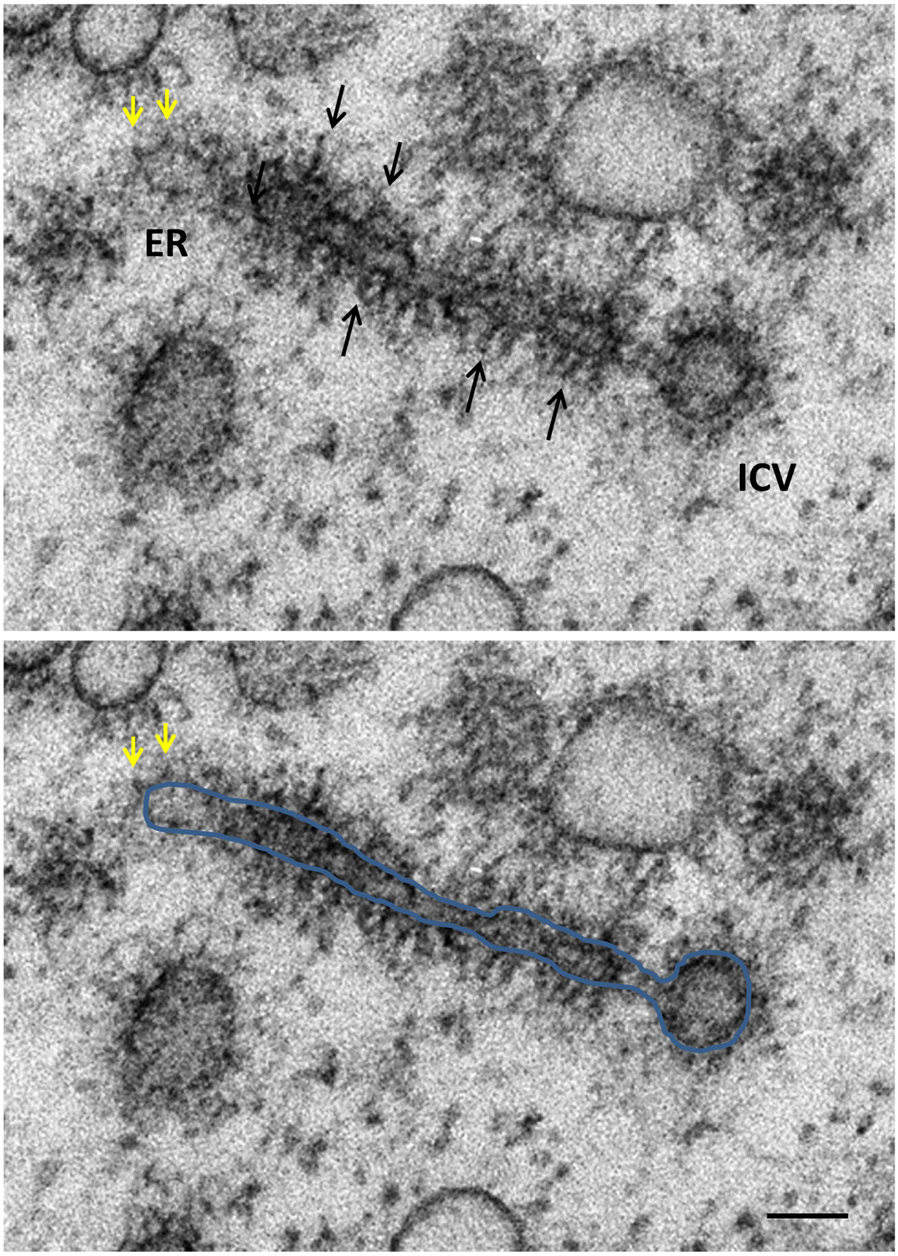

Supplementary Figure 15. ICVs can bud from remodeled ER (I).
Images are enlargements of Figure 7B and show a thin ER cistern covered by vRNPs-like filaments (black arrows). A few ribosomes (yellow arrows) are still attached to the structure whose membrane is continuous with the budding ICV as highlighted with a blue line in the bottom panel.
Scale bar, 100 nm.

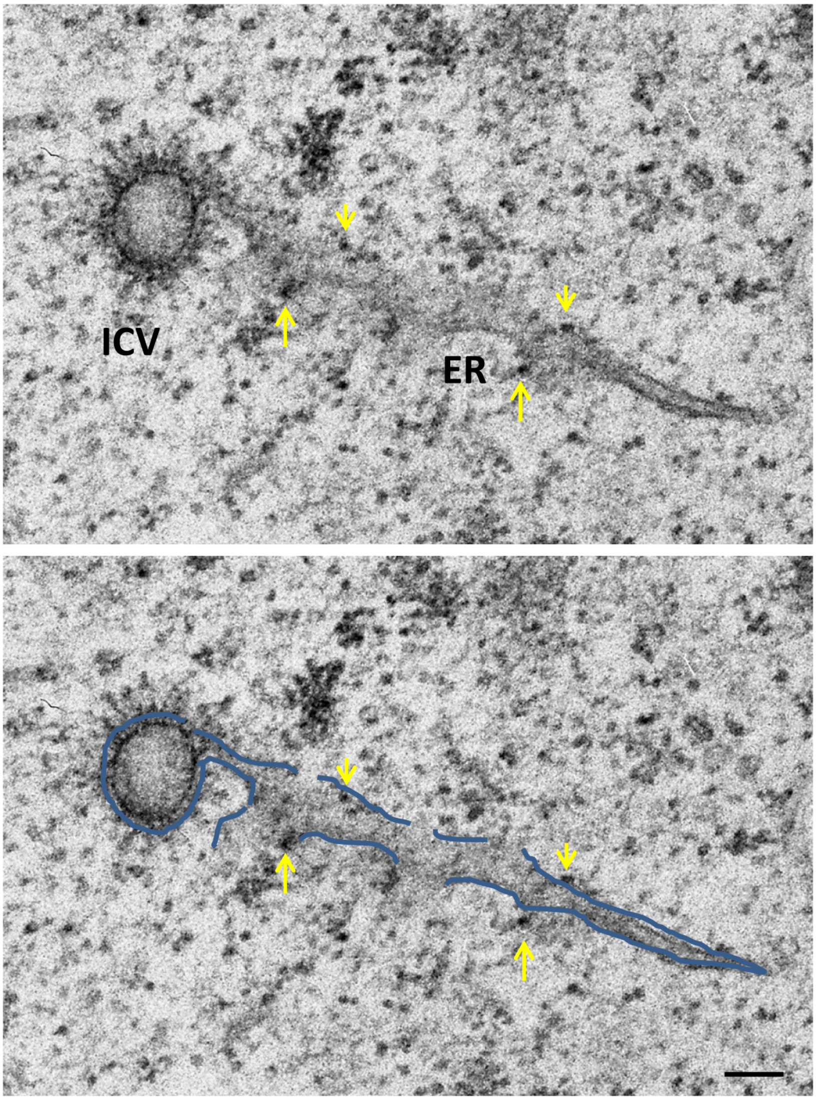

Supplementary Figure 16. ICVs can bud from remodeled ER (II). Images are enlargements of Supplementary Fig. 4C and show an ICV budding from an ER cistern. Continuity between the remodeled ER element and the budding ICV Is highlighted with a blue line in the bottom panel. Ribosomes are marked with yellow arrows. Scale bar, 100 nm.

A

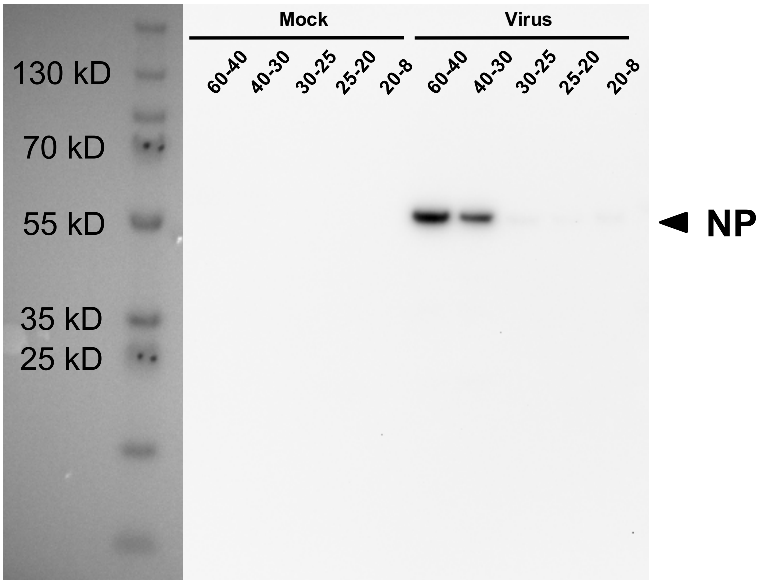

B

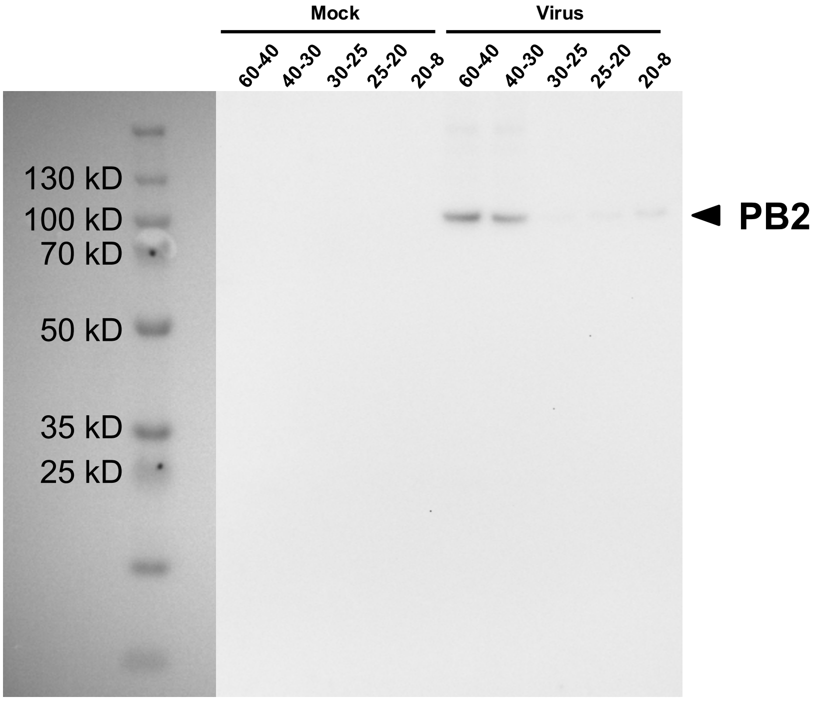

Supplementary Figure 17. Western-blot analysis of subcellular fractions of mock-infected (left panel) and IAV-infected (right panel) A549 cells, upon sucrose gradient ultracentrifugation.

A549 cells were infected with the WSN virus and submitted to subcellular fractionation at 14 hpi. The same amount of total protein was analyzed for each fraction, using (A) an anti-NP antibody, (B) anti anti-PB2 antibody.

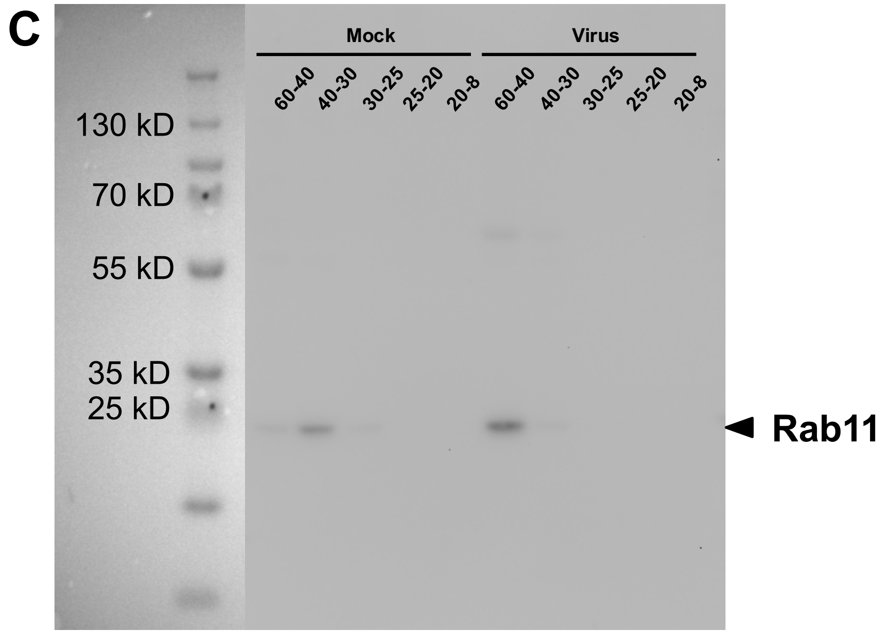

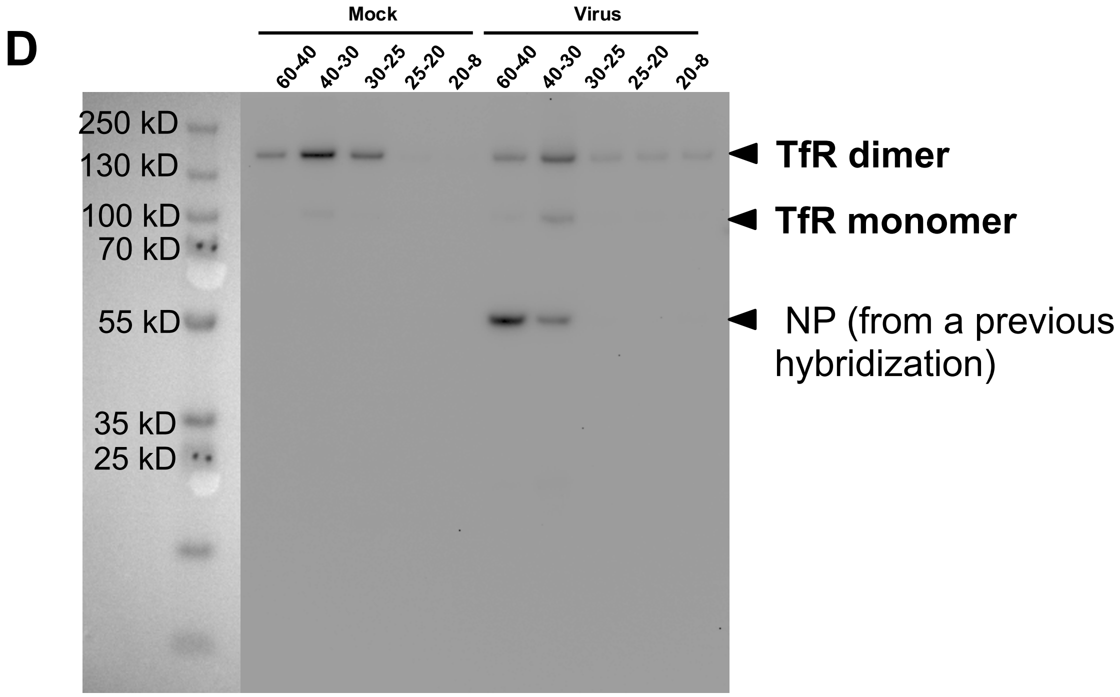

Supplementary Figure 17 (continued). Western-blot analysis of subcellular fractions of mock-infected (left panel) and IAV-infected (right panel) A549 cells, upon sucrose gradient ultracentrifugation. The same amount of total protein was analyzed for each fraction, using (C) an anti-Rab11 antibody, (D) an anti-transferrin receptor antibody. In our conditions, most of the transferrin receptor was detected as a dimeric complex (expected MW of the monomer and homodimer : 95 and 190 kD, respectively).

E

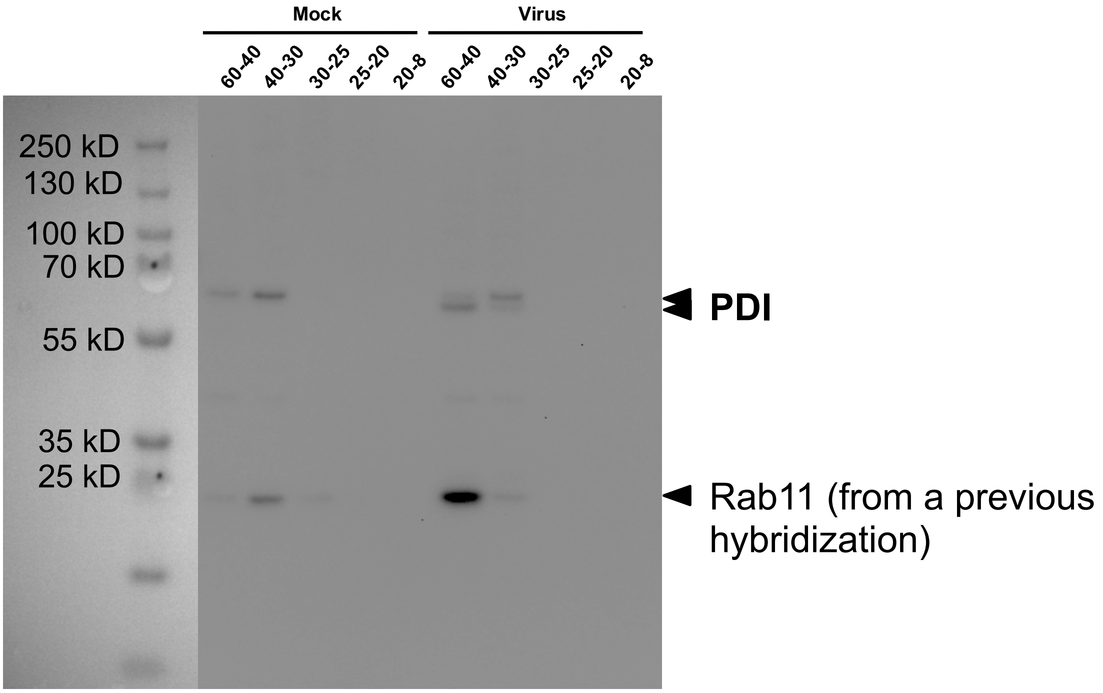

F

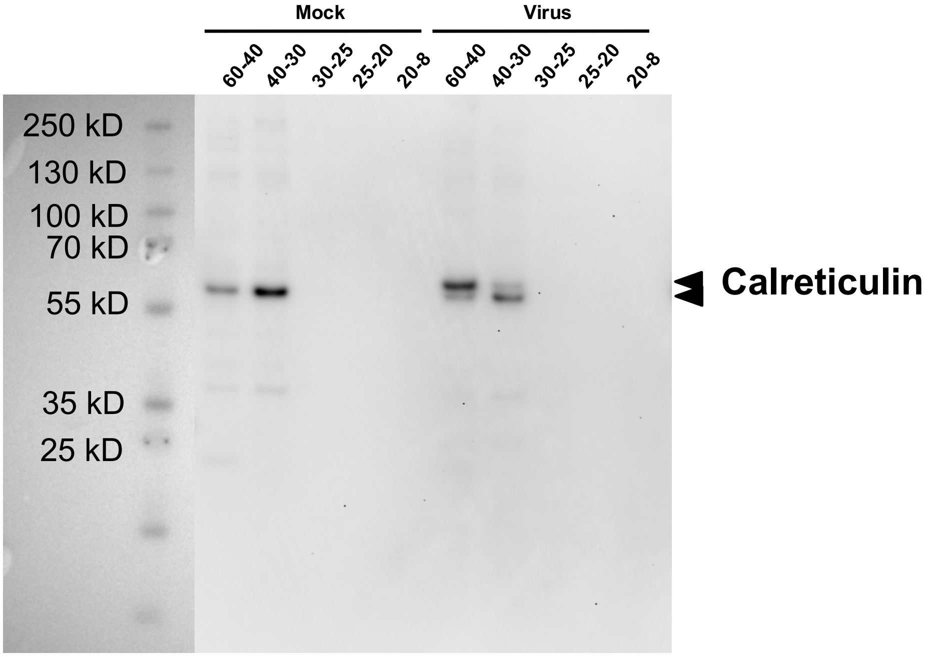

Supplementary Figure 17 (continued). Western-blot analysis of subcellular fractions of mock-infected (left panel) and IAV-infected (right panel) A549 cells, upon sucrose gradient ultracentrifugation. The same amount of total protein was analyzed for each fraction, using (E) an anti-Protein Disulfide Isomerase antibody, (F) an anti-calreticulin antibody.

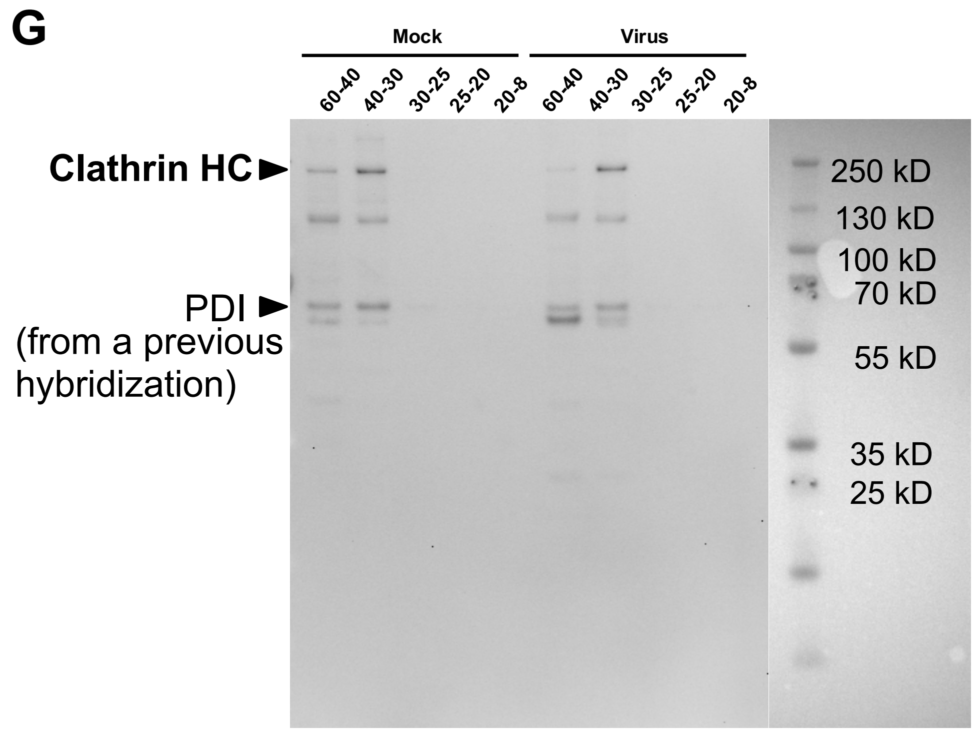

Supplementary Figure 17 (continued). Western-blot analysis of subcellular fractions of mock-infected (left panel) and IAV-infected (right panel) A549 cells, upon sucrose gradient ultracentrifugation. The same amount of total protein was analyzed for each fraction, using (G) an anti-clathrin heavy chain antibody.

Supplementary Table 1: Sub-cellular distribution of ICVs in IAV-infected A549 cells at 8 hpi

	ICVs	
**Cell #**	**MTOC area[a]**	**Other area[a]**
1	26	6
2	42	12
3	99	12
4	37	8
5	64	14
6	34	11
7	29	15
8	28	3
9	20	3
10	15	4

[a] as defined in the diagram below

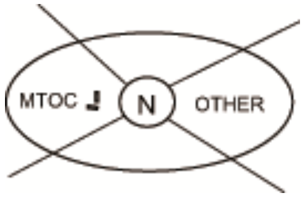

Supplementary Table 2: Sub-cellular distribution of groups of vRNPs in IAV-infected A549 cells at 8 hpi

	Groups of vRNPs				
**Cell #**	**ne[a]/nuclear pores**	**ER**	**Filaments**	**ICVs**	**Cytosol**
1	19	16	0	14	6
2	12	22	1	6	5
3	21	18	6	5	2
4	4	9	0	6	3
5	5	9	0	4	2
6	6	15	0	6	3
7	10	17	0	8	4
8	4	13	2	7	5
9	4	9	0	7	2
10	8	13	0	8	3
11	7	9	3	4	0
12	12	20	4	5	0
13	4	7	0	10	3
14	3	5	0	8	1
15	8	11	5	16	4
16	6	8	3	12	0
17	10	6	0	4	3
18	9	5	2	7	5
19	8	7	0	5	1
20	7	10	2	5	4
21	2	10	0	3	0
22	15	9	0	7	0
23	7	6	0	3	2
24	8	11	0	4	1
25	10	6	0	5	0
26	5	6	0	7	3
27	10	7	0	9	1
28	9	5	0	10	4
29	6	4	3	8	5
∑	**239**	**293**	**31**	**203**	**72**

[a] ne: nuclear envelope

Supplementary Table 3: Sub-cellular distribution of groups of MT-Rab11-wt or MT-Rab11-S25N in IAV-infected A549 cells at 8 hpi

	Groups of Rab11							
	Rab11-wt				Rab11-S25N			
Cell #	ne[a]	ER	ICVs	Smooth vesicles	ne	ER	ICVs	Smooth vesicles
1	0	5	7	0	0	0	0	4
2	0	9	8	0	0	0	0	6
3	3	7	9	0	0	3	0	5
4	0	5	5	0	0	0	0	7
5	0	6	7	0	0	0	0	3
6	0	10	8	0	0	5	0	5
7	0	8	4	0	0	0	0	4
8	0	11	3	0	0	0	0	6
9	0	5	7	0	0	0	0	2
10	2	12	5	0	0	4	0	3
11	0	4	9	0	0	0	0	4
12	4	2	3	0	0	0	0	7
13	0	8	0	0	0	0	0	2
14	5	7	10	0	0	0	0	5
15	0	4	6	0	0	3	0	0
16	0	0	5	0	0	0	0	7
17	2	8	4	0	0	0	0	4
18	0	3	8	0	0	5	0	3
19	0	9	7	0	0	2	0	6
20	0	10	6	0	0	0	0	5
21	1	7	4	0	0	0	0	2
22	2	4	8	0	0	0	0	1
23	0	5	5	0	0	0	0	4
24	0	6	3	0	0	1	0	3
25	0	3	2	0	0	2	0	5
∑	**19**	**158**	**143**	**0**	**0**	**25**	**0**	**103**

[a] ne: nuclear envelope

Supplementary Table 4: Distance of ICVs from the plasma membrane on n=25 IAV-infected A549 cells at 8 hpi.

	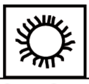	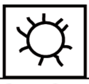	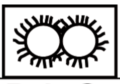
**Distance from pm[a]**	**Single Filaments +++[b]**	**Single Filaments +[b]**	**Pairs[c] Filaments +++[b]**
**< 200 nm**	51	58	7
**> 200 nm**	57	5	21

[a] pm: plasma membrane

[b] +++: ICVs with a dense filament coat; +: ICVs with a sparse filament coat

[c] ICVs in pairs always show a dense filament coat
